# Bayesian Estimation of Animal Movement from Archival and Satellite Tags

**DOI:** 10.1371/journal.pone.0007324

**Published:** 2009-10-13

**Authors:** Michael D. Sumner, Simon J. Wotherspoon, Mark A. Hindell

**Affiliations:** 1 Michael D. Sumner School of Mathematics & Physics (IASOS), University of Tasmania, Hobart, Tasmania, Australia; 2 Simon J. Wotherspoon School of Mathematics & Physics, University of Tasmania, Hobart, Tasmania, Australia; 3 Mark A. Hindell School of Zoology (Antarctic Wildlife Research Unit), University of Tasmania, Hobart, Tasmania, Australia; Lund University, Sweden

## Abstract

The reliable estimation of animal location, and its associated error is fundamental to animal ecology. There are many existing techniques for handling location error, but these are often ad hoc or are used in isolation from each other. In this study we present a Bayesian framework for determining location that uses all the data available, is flexible to all tagging techniques, and provides location estimates with built-in measures of uncertainty. Bayesian methods allow the contributions of multiple data sources to be decomposed into manageable components. We illustrate with two examples for two different location methods: satellite tracking and light level geo-location. We show that many of the problems with uncertainty involved are reduced and quantified by our approach. This approach can use any available information, such as existing knowledge of the animal's potential range, light levels or direct location estimates, auxiliary data, and movement models. The approach provides a substantial contribution to the handling uncertainty in archival tag and satellite tracking data using readily available tools.

## Introduction

Estimating the movements of animals is a fundamental requirement for many ecological questions. These include elucidating migratory patterns, quantifying behavior in terms of the physical environment and understanding the determinants of foraging success, all of which can influence larger population processes [1]–[3]. Types of movement data can range from simple mapping of positions to behavioral models that attempt to account for unlikely estimates, provide estimates of behavioral states and predict latent variables.

There are two common methods for obtaining position estimates, which can be broadly categorized as remote and archival. Remote methods use techniques such as radio or satellite telemetry to locate a tag attached to an animal. Archival methods require the tag to record aspects of the animal's environment over time (such as light levels and water temperature) which are then processed to infer location [1], [4], [5].

Before any analysis can be done, position estimates require some quantification of precision and accuracy to provide statistical confidence in results [6]–[8]. Quantification of location precision, and crucially, the incorporation of these into synoptic spatial representations of animal movement, is an important problem common to both methods that many authors have attempted to address in recent studies [9]–[14].

Location precision is generally lower in archival methods due both to the theoretical basis and practical problems of the location estimation [15], [16]. To overcome this limitation, archival methods routinely integrate primary location estimation with auxiliary data sets [4], [12], [17], [18]. In principle this enables the integration of the estimation and error estimation processes but this remains an under-utilized opportunity: published uses of archival methods usually separate the estimation of the quality of position estimates from their derivation. Satellite-derived estimates provide less opportunity in this regard, as the process is proprietary and information regarding error is minimal. However, satellite locations still require a modeling framework to incorporate auxiliary information and provide the best possible estimates [11] including a quantification of precision.

The simplest analysis of movement data is to visualize the sequence of locations visited by the animal. It is slightly more complex to provide a path estimate of the animal, which requires the ability to determine position both from available data as well as for latent times where no data were measured. An obvious simple model is to “join the dots”, assuming that movement is both linear and regular between measured positions. A more realistic approach demands that estimates of an animal's path consider both direct and latent location estimates, because in general there are open-ended scenarios that could occur between direct estimates. There are a multitude of methods for achieving this [14], [19]–[22], but none have been directly integrated with the estimation process from raw data.

Once an estimate of an animal's path is obtained biologists often need to calculate speed of and distance of travel, generate spatial representations of an animal's use of space in terms of time spent in geographic regions, metabolic effort or other measure of resource allocation. More sophisticated analyses aim to determine behavioral states more exactly [11], [23], or to differentiate migration from foraging behavior. These aims are beyond the present work, where we will be focusing on the first step in the process—description of an animal's path and the precision with which this can be estimated.

Earlier work has attempted to account for spatial uncertainty by choosing a scale for interpreting location data [14], or spatial smoothing [24]. These techniques fail to estimate statistical uncertainty for individual estimates, and provide only an overall average of precision. Other techniques are used to estimate latent position by interpolation or similar technique [21], but these must assume that positions are known.

Given the diversity of questions asked of movement data, there are understandably many approaches to data analysis. Many existing techniques are specific to particular questions and species and have little scope outside the given application. Further, each application has its own problems of scale, location error, data quality and summarizing of behavior. In this context, sophisticated model approaches are seeing greater use in tracking studies [13], but these have only been applied to pre-derived positions and leave the problem of location estimation from raw data unaddressed. No study has yet provided a general approach to dealing with the twin issues of estimate precision and accuracy for both archival and satellite location data. There is a growing need for just such an approach as more large multi-species studies are being undertaken [25]–[27]. Such multi-species studies inevitably utilize a range of tracking techniques as no one method is suitable for all species. For example, fish which rarely come to the surface are not usually suitable for satellite tracking [28].

Here we present a Bayesian framework for the analysis of movement data that directly addresses the estimation of location from raw data collected by archival tags and can also be applied to other datasets of pre-derived position estimates such as Argos locations. We apply the approach to both an archival tag dataset and a satellite tag dataset. Our primary goal is to integrate all available sources of information for estimating location. Using all available information may sound obvious, but it is a missed feature of many applications. Secondarily, we aim to integrate the location estimation and the estimation of location precision. The approach should also be able to provide all of the desired end-uses of tracking data as mentioned above. In the Bayesian context, each of these measures, including appropriate confidence intervals (CI) [29], [30], can be determined by specifying appropriate priors and distributions for each data source and calculating the posterior.

## Materials and Methods

### Ethics Statement

Data were collected under permits from the University of Tasmania Animal Ethics Committee (A6790 and A6711).

### Assumptions

We propose a Bayesian approach to the tag location problem that uses Markov Chain Monte Carlo methods to approximate the posterior.

There are three main elements to the process of Bayesian estimation; the prior, the likelihood and the posterior. The *prior* distribution 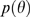 represents our knowledge of the parameters 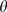 before any data is observed. The *likelihood* 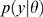 is the probability of observing data 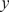 for a given set of parameters 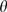, and represents our knowledge of the data collection process. From these we calculate the posterior distribution 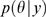 via Bayes' rule
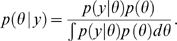
(1)


The posterior 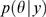 represents our knowledge of the parameters after the data 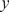 have been observed. In essence, Bayes' rule provides a consistent mechanism for updating our knowledge based on observed data.

The data available for forming location estimates can be classified into four broad types.

#### Prior knowledge of the animal's movements

Invariably something is known of an animal's home range, migratory pattern or habitat preference, and any location estimate should be consistent with this information. This information can range from being quite specific such as the species generally stays over the continental shelf (e.g. shy albatross [31]) or more vague such as the species often heads south (e.g. southern elephant seals [32]).

#### Primary location data

The primary location data 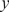 is data collected primarily for the purposes of location estimation, and directly inform about the locations 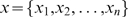 of the tag at a sequence of (possibly irregular) times 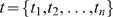. Examples include the light levels recorded by an archival tag, or for an Argos tag the locations provided by the Argos service.

#### Auxiliary environmental data

Many tags also record additional environmental data 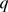, and this data may be compared to external databases to further constrain location estimates [4], [12], [13], [17], [18]. For example, in the marine context depth and temperature measurements can be compared to remotely sensed or modelled sea surface temperature (SST) data to confine locations to regions where SST is consistent with the temperatures observed by the tag.

#### Movement models

Movement models constrain the trajectory of the animal, reducing or removing the occurrence of location estimates that correspond to improbable or impossible trajectories. Several forms of movement models appear in the literature; at the simplest level is speed filtering which prohibits estimates that imply impossible speeds of travel [33], [34], while other authors propose more complex state space approaches that model correlation between successive legs of the trajectory [11], [23].

Several authors have noted the advantages of Bayesian methods in complex problems in ecological research [35]–[39]; for the tag location problem one principal advantage is that four disparate data sources can be systematically incorporated into a single unified estimator of location.

The novel aspect of the method we propose is the adoption of a simple yet powerful representation of the movement model that not only constrains the animal's trajectory, but also allows this trajectory to be estimated. Between each pair of successive locations 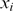 and 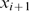, introduce a new latent point 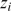 representing the location of the tag at a time 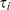 uniformly distributed in the interval 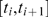, and let 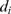 be the length of the dog-leg path from 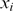 through 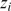 to 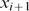. The movement model then simply prescribes the joint distribution 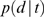 of the dog-leg distances 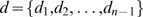. For example, adopting a model where the 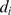 are independently uniformly distributed
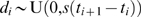
implements a simple speed filter that limits the maximum speed of travel to 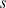. Alternately, migration and large scale consistency of motion can be modelled by adopting a distribution that allows for more complex patterns of dependence between the successive 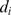.

Note there is no explicit expression for the 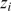, they are defined implicitly through the dog-leg distances 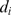. However, any choice of 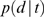 that places realistic bounds on each 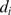 is sufficient to ensure that the 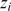 are estimable (in a Bayesian sense), while also constraining location estimates. Most importantly, as 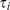 is uniformly distributed in the interval 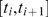, the posterior distribution for 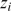 describes the possible paths between 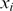 and 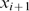. In a sense, 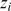 is not intended to refer to the tag location at one particular time in the interval 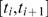, but all times in the interval 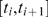.

The second key assumption of the method is that the primary location data, the auxiliary environmental data and the behavioural model are all independent, and so the likelihood 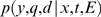 reduces to a product of contributions from each of these three sources
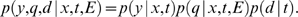



Here 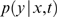 is the likelihood of observing the primary location data 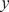 given locations 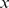 at times 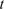, 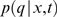 is the likelihood of observing the environmental data 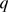 given locations 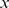 at times 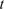 and a database 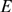 of known environmental data, and 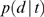 is the distribution of dog-leg distances between the successive locations described above. The exact form of 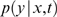 and 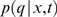 will depend on the precise nature of the data collected by the tag, and several common examples are discussed below.

The prior for 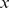 and 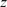 reflects knowledge of the animal's home range, habitat preference, migratory patterns or other fundamental environmental considerations. For example, a known home range can be modelled by adopting a prior of the form
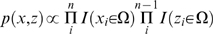
where 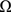 is the known home range and 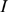 is the indicator function
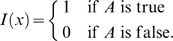



Migration can be accommodated by allowing 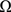 to vary with season, while habitat preference can be incorporated by assigning greater probability density to more favourable habitat. We must also supply a prior for 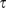 that simply reflects our assumption that 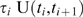. The form of 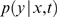 as the contribution of the primary location data to the total likelihood depends on the nature of the tag in question.

### Satellite tags

For satellite tracked tags, the primary location data 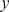 consists of direct estimates 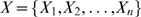 of the true tag locations 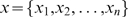 at times 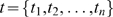 provided by a remote sensing service, possibly augmented with some indicators of location reliability 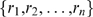. In this case the contribution 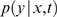 to the total likelihood is determined by assuming the observed locations 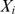 are bivariate Normally distributed about the true locations 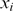,
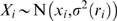
with a variance 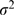 that is a function of the reliabilities 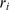. For less consistent services, longer tailed distributions such as the bivariate 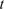 can be used to accommodate the occasional erroneous location [29].

### Archival tags

For archival tags there are no initial estimates of tag location; the primary location data consists of light intensities recorded by the tag at regular intervals over the day. The tags' location can be estimated from the light level data by the methods of [40] and [15]. We use a version of the template-fitting method [40] to provide a location estimate for each twilight. The full computational details are complex and will be the subject of a future publication, but in essence the method is as follows. The time series of light levels corresponding to each twilight recorded by the tag is extracted, and for marine applications, corrected for attenuation due to depth. This yields a sequence of time series; one time series 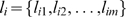 for each twilight, where 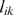 is the corrected light level recorded at time 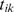. A function 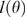 that maps solar elevation 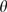 to the (unattenuated) log light level 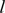 recorded by the tag is determined by laboratory calibration. The contribution 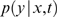 to the total likelihood is determined by assuming the log corrected light levels are distributed as
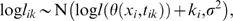
where 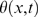 is the Sun's elevation at location 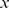 and time 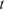, and 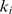 is a constant to allow for attenuation due to cloud. The variance 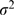 is determined by the recording error in the tag.

Similarly, the contribution 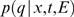 the auxiliary environmental data 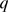 makes to the total likelihood will depend on the nature of the data recorded by the tag and the availability of a suitable reference database 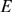 with which to compare.

For example, for marine tags that record both water temperature and depth, for each 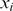 an estimate 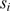 of the SST can be derived from the temperature and depth data recorded by the tag in some small time interval 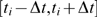 surrounding 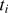. This estimate might then be assumed to be Normally distributed about a reference temperature 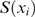 determined from a remotely sensed SST database 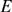,
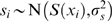
where the variance 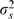 is determined by the accuracy of both the tag and the remotely sensed database. Alternately, a more conservative approach similar to that employed by [41] is to suppose that the temperature 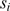 measured by the tag is a very poor indicator of average SST, but could be no greater than an upper limit 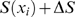 and no lower than 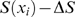 and assume 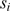 is uniformly distributed in this interval
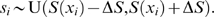



Again 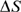 is determined by both the accuracy of the tag and database.

As a second example, for marine applications the depth data recorded by a tag can be exploited by noting that the maximum depth recorded in a time interval 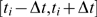 surrounding 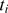 provides a lower bound 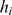 for the depth of the water column at 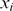. We can then refine the estimate of 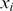 comparing 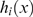 to a high resolution topography database 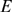 and excluding regions that are too shallow by including in the likelihood a factor of the form
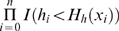
where 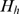 is the bottom depth determined from the database and 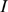 is again the indicator function.

### Posterior estimation

Once the prior and likelihood have been defined, the posterior 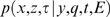 is determined by Bayes' rule
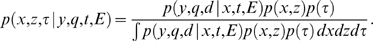



Typically however, the integral in the denominator is computationally intractable, and instead we resort to Markov Chain Monte Carlo (MCMC) to approximate the posterior.

MCMC [30] is a family of methods that allows us to draw random samples from the posterior distribution. Summarizing these samples approximates the properties of the posterior, in the same way that a sample mean is an approximation to a population mean. In principle, the approximation can be made arbitrarily accurate by increasing the number of samples drawn.

For the tag location problem we use a block update Metropolis algorithm based on a multivariate Normal proposal distribution [30]. The Metropolis algorithm was chosen for its simplicity and genericity – it is easily implemented and the implementation is not strongly tied to particular choices of likelihood and prior. We have used a block update variant of the algorithm, where each 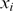 and each 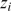 are updated separately. Using a block update improves computational efficiency provided parameters from separate blocks are not strongly correlated. For the time intervals between locations typical of satellite and geolocation data and reasonable choices of movement model 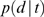, we have not found the correlation between successive locations estimates to be so great as to greatly impede the mixing of the chain.

### Examples

To illustrate this basic framework, we present two simple examples.

The first example is a Weddell seal tagged at the Vestfold Hills (78
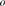
E, 68
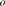
S) tracked with a satellite tag (9000X SRDL; Sea Mammal Research Unit, St. Andrews, Scotland) with locations provided by the Argos service [42].

The Argos service provides approximate locations 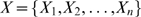 and corresponding location qualities 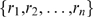 for a sequence of times 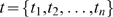. This forms the primary location data. Each 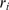 categorizes the corresponding 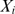 into one of seven quality classes based on the number of satellites used in its determination [42]. We translate the 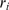 into approximate positional variances 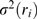 based on the results of [43] and assume
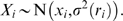



So that the contribution to the likelihood from the primary location data is
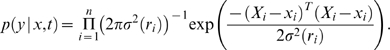



This particular tag recorded no environmental data, and so the corresponding contribution to the likelihood is 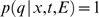.

For this example a very simple movement model was adopted. We choose 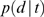 so that the mean speeds 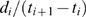 between successive locations are independently log Normally distributed
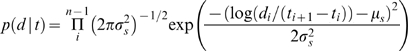
with 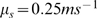 and 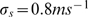, where these figures were chosen conservatively based on an examination of Argos data of the highest quality class.

Finally, we adopted a prior 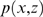 for 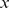 and 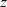 that was uniform over the ocean, that is
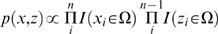
where 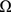 is the ocean. This was implemented by comparing 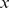 and 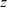 to a high resolution land/sea raster mask generated from A Global Self-consistent, Hierarchical, High-resolution Shoreline Database [44]. Creating a raster mask to indicate sea/land allows the prior to be computed very efficiently by avoiding complicated point-in-polygon tests.

The second example is a mature southern elephant seal (*Mirounga leonina*) tagged at Macquarie Island (158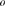 57′E, 54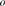 30′ S), with data from a time-depth-recorder (Mk9 TDR; Wildlife Computers, Seattle, WA, USA). The data were collected using methods described by [45]. This tag provides regular time series of measurements of depth, water temperature, and ambient light level.

In this case the primary location data consist of the time series of depth and ambient light level. As outlined above, the depth adjusted light level is assumed to be log Normally distributed about the log expected light level for the sun elevation adjusted for cloud cover so that
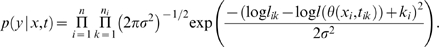



For this example, the depth and water temperatures recorded by the tag were used to estimate sea surface temperatures that were then compared to NCEP Reynolds Optimally Interpolated SST. For each twilight, estimates of minimum 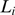 and maximum 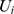 SST observed in the surrounding 12 hour period were derived from the depth and water temperature records. These estimates form the auxiliary environmental data 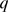, and 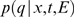 was then chosen as
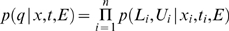
where
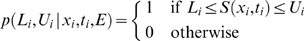
and 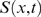 is the NCEP Reynolds Optimally Interpolated SST. This example shows the great difficulty in choosing 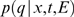 – typically the data from the tag and the data from the reference database are recorded on wildly disparate spatial and temporal scales, making it very difficult to make any reasonable comparison of the two.

Again the movement model 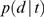 is chosen so that the mean speeds 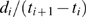 between successive locations are independently log Normally distributed
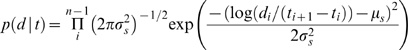



In this case we use 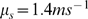 and 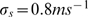, and these figures were chosen conservatively based on knowledge of elephant seal behaviour.

Finally, just as for the satellite tag example a prior 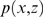 uniform on the ocean was adopted 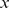 and 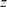, but in this case the land/sea raster mask generated from the 2-Minute Gridded Global Relief Data (ETOPO2).

The primary rationale behind our choices for examples was to show the application of our approach to both satellite locations and archival tag data. Further to this, for the satellite example we wish to demonstrate the use of our approach for a situation involving a complex inshore coastline and the handling of existing estimates that occur on land. We are not attempting to show the best possible application for our examples, but demonstrating a consistent approach that is able to use all available sources of data.

## Results

For the satellite tag example an initial 10,000 samples were drawn and discarded to allow for both burn-in and tuning of the proposal distribution [30]. A further 300,000 samples were then drawn, and standard convergence tests applied [46]. The same strategy was adopted for the archival tag example, with 30,000 samples drawn for burn-in, and a further 800,000 samples drawn. In neither case was there any evidence that the chains had failed to converge, but it must be realized that these are problems of extremely high dimension, and as such a subtle convergence problem may be difficult to detect.

**Figure 1:**
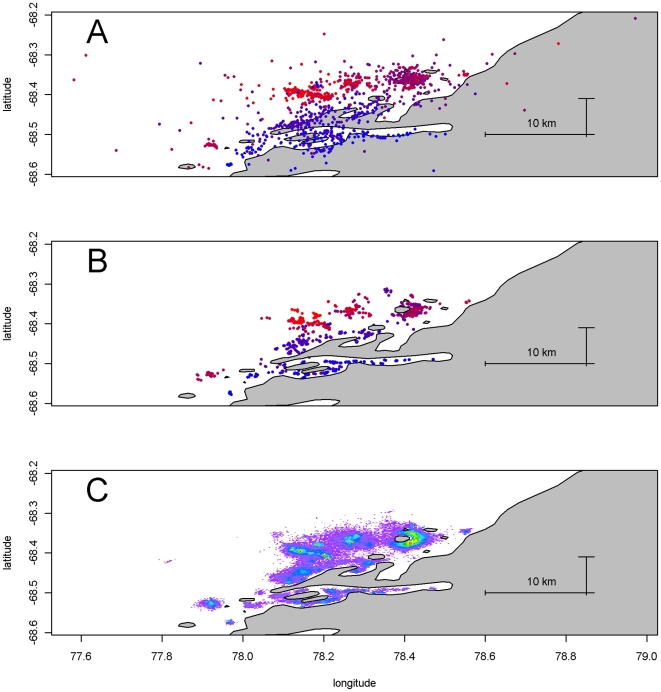
Satellite tag data and estimates. Panel A: The sequence of original Argos estimates for an adult female Weddell seal tagged in the Vestfold Hills, with time scale from red to blue. All location classes are shown. The different length scale bars for north and east represent 10 kilometers. Panel B: Posterior means for 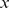 from the Argos dataset plotted spatially, with time scale from red to blue as in panel A. The sequence is far more realistic, without the noise and positions on land. Panel C: Map of time spent from full path estimates from the Argos dataset. The density represents a measure of time spent per area incorporating the spatial uncertainty inherent in the model. Bin size is 150 m by 140 m.

**Figure 2:**
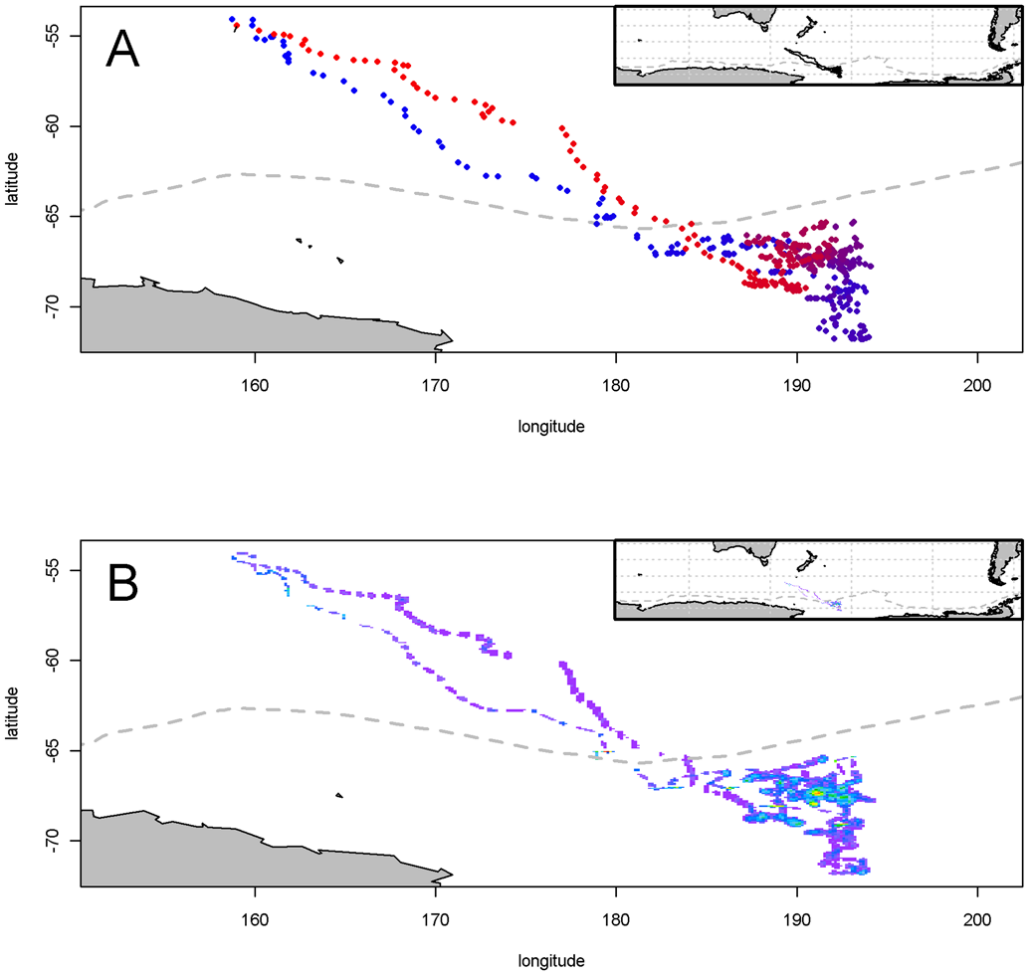
Estimates and time spent for archival dataset. Panel A: Posterior means for 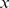 from the archival dataset plotted spatially, with time scale from red to blue. The sequence provides a realistic trajectory for an elephant seal. The dashed grey line shows the (approximate) position of the Southern Boundary of the Antarctic Circumpolar Current. Panel B: Map of time spent from full path estimates from the archival dataset. Bin size is 5.5 km by 9.3 km at 54 S and 3 km by 9.3 km at 72 S.

The provided Argos Service locations for the satellite tag example are displayed in Figure 1a, showing the primary location data. This includes all raw positions from Argos, including every location quality class. The time-series of locations, is quite noisy and many of the positions fall on land. The sequence suggests that the animal has begun in the southern region of the area, with excursions into and out of various inlets, traveling to the north overall, but with an excursion returning to the south somewhat offshore. The record ends in the northern region. From this plot it is clear that there are many unlikely locations given the presence on land and the implied tortuous path. The outputs of our modelled estimates for this data set are discussed below. Posterior mean locations for 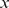 from the archival tag dataset may be seen in Figure 2a. Unlike the Argos example, there are no ‘raw locations’ to present as the primary location data are light level measurements. The range of the track estimate has no local topographic features (coastline or bathymetry) that constrains the locations, as the area visited is for the most part deeper than −2000 m [44]. However, we know that these locations are consistent with the matching sea surface temperature data, under the assumptions of our model.

### Argos tag dataset

**Figure 3:**
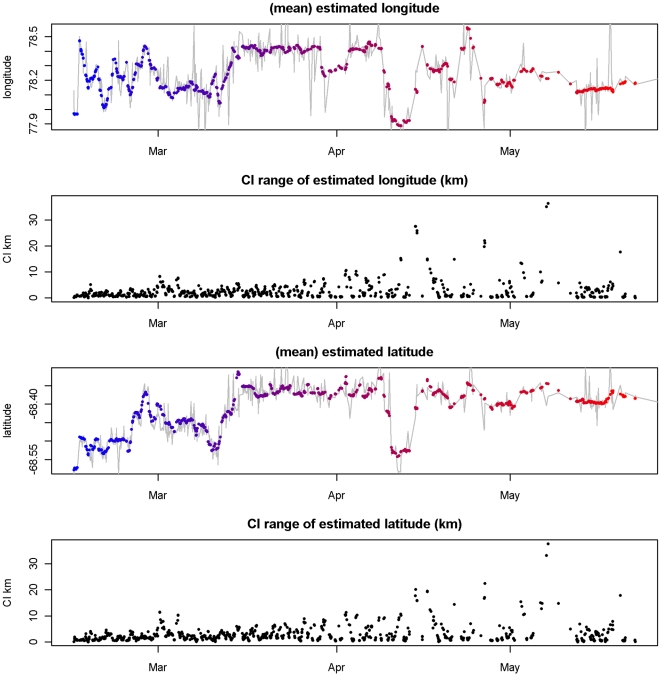
Individual longitude, latitude estimates for Argos. Posterior means for 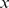 from the Argos dataset for longitude and latitude, with time scale from red to blue as in Figure 1. The grey line shows the implied sequence of the original Argos estimates. Also shown is the range of the 95% CI of each estimate (km), determined with the mean by directly summarizing the posterior.

In Figure 3 the posterior means for 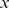 are plotted separately for longitude and latitude with the sequence of original Argos Service positions overplotted as a line. Also shown are the individual confidence interval (CI) estimates (95% level, presented as a range in kilometers). The sequence of estimates is clearly more realistic than the original Argos locations in terms of likely movement, even though no time steps have been discarded. The confidence intervals in Figure 3 are summarized from their 2-dimensional versions and plotted here with longitude and latitude separated to easily show the relative precision of each. Most of the estimates have a range of less than 5 km, with a maximum above 30 km. This simple plotting of individual parameters with CIs leaves out a lot more information than exists in two dimensions. A supporting information file (Figure S1) provides an animation of the full path with the implied path of the original Argos locations to illustrate the improvement provided by our approach. The posterior means for 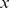 longitude and latitude are presented spatially in Figure 1b. The main differences with the raw estimates is that there are now no estimates that fall on land, and the sequence of positions is far more realistic in terms of likely movement. The 1124 original Argos locations included 179 that fell within the bounds of the coastline data used. The overall travel to the north can be seen in more detail, with an excursion into the main large inlet and then movement around the bay into the region of islands to the north. There are two large excursions when the animal has returned briefly to the southern region, first to the large inlet, then to an island further south, but the more extreme outliers are no longer present. This journey is typical for these seals, as shown by [47]. (We do not present the points connected by lines as this would be visually messy and also imply impossible trajectories based on the simplistic “join the dots” model. The connectivity, or full-path, of estimates is provided by the intermediate estimates.) A map of time spent per unit area is shown in Figure 1c. This density plot shows the “full path” estimate using the intermediate locations, summarized by binning the posterior and weighting each segment by the time difference between each original Argos time step. The full track estimate is shown here providing a single view of the entire trip. Again, this neglects a lot of information that is available from the posterior, as any segment of the path may be interrogated, down to the level of individual estimates. The bin size here is 150 m by 140 m, simply chosen for convenience given the image plot size. This image portrays the areas of most time spent by the animal, with the spatial precision of estimates implicit in the spread of time-spent density. Importantly, the transition between time in the water and the position of land is smooth as the estimation takes the presence of land into account as it proceeds. There is no artificial clipping of the distribution as would be required if a simple spatial smoother was used on raw estimates. This achieves the shared goals of smoothing techniques such as kernel density [48] and cell gridding.

A summary of the precision of estimates for longitude and latitude for each original Argos class estimate is presented in Table 1. This summary shows that our estimates are consistent with and often better than the expected precision given by the Argos class and, while that point is slightly circular given our use of the class information in the model, our approach is able to combine the contribution of the Argos class with other information and show that the precision of estimates is not necessarily directly related to the class assigned.

**Table 1:** Estimate precision for Argos dataset.

Longitude					
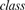	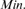	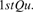	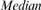	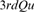	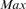
Z	0.27	1.09	1.90	2.99	22.05
B	0.27	0.95	1.77	3.95	36.20
A	0.27	1.09	2.18	3.78	15.38
0	0.13	1.36	2.30	4.08	25.86
1	0.27	0.82	1.23	2.04	5.99
2	0.14	0.41	0.61	0.95	2.31
3	0.14	0.27	0.41	0.54	1.50
Latitude					
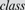	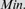	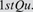	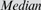	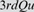	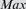
Z	0.45	1.21	1.97	3.79	17.13
B	0.15	1.21	2.12	4.40	37.75
A	0.30	1.52	2.27	4.40	13.64
0	0.15	1.52	2.50	4.66	19.56
1	0.15	1.06	1.67	2.73	14.86
2	0.15	0.60	0.99	1.67	5.00
3	0.15	0.45	0.61	1.06	3.03

Summary of precision calculated from the posterior for 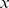 by original Argos class (km). Each row presents a quantile summary for the CI ranges (95%) from each Argos class for longitude and latitude. The seven classes are an attribute provided with the original Argos locations [42].

**Figure 4:**
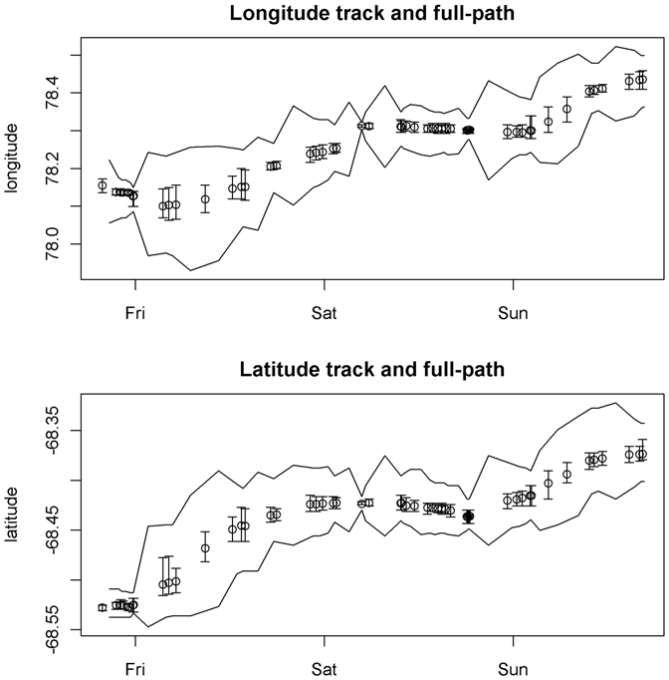
Intermediate estimates for Argos Posterior means for 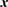 of longitude and latitude for a short period (23–26 Feb 2006) with CI ranges shown. The CI range for intermediate estimates (full path) is shown as a continuous band.

Finally in Figure 4 we can see the relationship between the direct estimates (plotted individually with CI ranges) and CI range of intermediate estimates (plotted as a continuous band) for a short period between 23–26 February 2006. The intermediate estimates provide a continuous path estimate, with latent times of no data “filled in” with estimates constrained only by the movement model and the environmental data. This figure also shows the utility of the method in terms of providing overall full path estimates, as well as individual point estimates with a measure of precision. Figure 4 also shows a deficiency of the assumed movement model - the estimated path at each 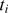 tends to be more variable than the corresponding 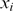. This is because there is no constraint on the individual legs of the dog-leg path from 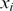 to 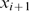. So it is possible for 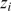 to be a great distance from 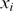 an instant after 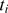 or from 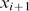 an instant before 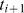, provided the total distance traversed over the dog-leg path is reasonable. It is difficult to resolve this issue without requiring a much more detailed understanding of the animal's behaviour.

### Archival tag dataset

**Figure 5:**
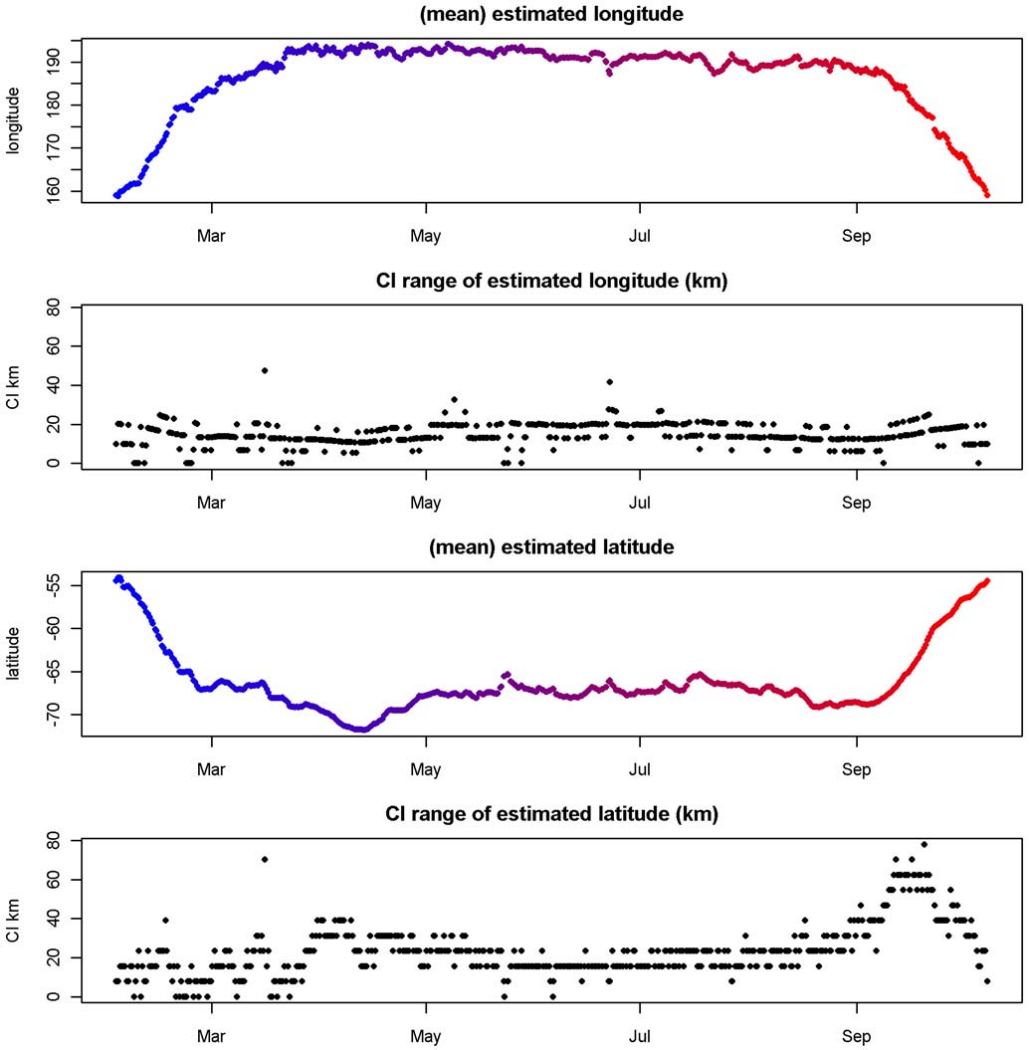
Posterior means for archival dataset. Posterior means for 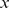 from the archival dataset for longitude and latitude, with time scale from red to blue as in Figure 2a. Also shown is the range of the 95% CI of each estimate (km), determined with the mean by directly summarizing the posterior.

Posterior means for 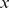 longitude and latitude are plotted separately with accompanying confidence intervals Figure 5. This includes a location for every local twilight, as seen in the raw light data. The sequence seems consistent with the time steps involved (12 hourly, on average), with no extreme or obviously problematic movements. The confidence interval of each estimate is also plotted, with a spatial range that is usually less than 30 km for longitude and 40 km for latitude. A summary of the precision of estimates for longitude and latitude is presented in Table 2.

**Table 2:** Estimate precision for archival dataset.

Longitude				
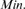	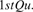	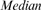	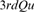	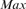
3.74	15.52	18.51	21.42	57.03
Latitude				
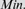	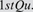	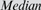	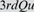	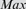
3.74	15.52	18.51	21.42	57.03

Summary of precision calculated from the posterior for 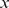 from the archival tag. A quantile summary for the CI ranges for longitude and latitude.

These estimated location are plotted spatially in Figure 2a. This animal has left Macqurie Island (1 February, 2005) and traveled directly to the southeast to a region north of the Ross Sea. Here it spends the period from early March to mid September with a short excursion to the south during April. Finally the animal reverses its outward journey, returning to Macquarie Island on 8 October 2005. The sequence of locations seems reasonable, with no obviously extreme estimates, and this is a fairly typical journey for these seals [32]. In Figure 2b a density map shows more clearly the spatial precision of the estimates and the areas where most time has been spent. It is clear that this region south of the Southern Boundary of the Antarctic Circumpolar Current [49] is an important feeding area for this animal.

**Figure 6:**
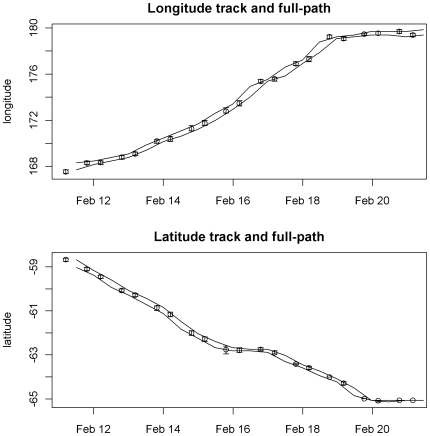
Intermediate estimates for archival dataset. Individual mean estimates of longitude and latitude for a 10 day period in February with CI ranges shown, as well as the CI range for intermediate estimates (full path) shown as a continuous band.

A summary of the precision of estimates for longitude and latitude is presented in Table 2. We can see the distinction between the direct and intermediate estimates plotted in Figure 6. This time the difference between the direct and intermediate estimates is less than with the satellite tag example.

## Discussion

The flexibility provided by Bayesian methods for complex problems [36], [38], [50] proved fruitful in this study. We have demonstrated a general approach for estimating true locations from both archival tag data and satellite fixes, accepting either source as raw data. This approach handles erroneous existing location estimates and other problems by incorporating all available sources of information in one unified process. We have shown how this approach can be used to obtain all of the common measures of interest in tracking studies by summarizing the posterior. These are path estimates, estimate precision, latent estimates, combinations and diagnostics of location estimates.

### Path

The likely (posterior mean) path for a basic representation of position over time. These can be used to plot simple tracks, or to query other datasets (such as productivity measures) for corresponding information at that location and time.

### Precision

For each estimate we can obtain precision estimates (CI). These probability densities are bivariate and can be obtained separately for each time step in the sequence, or for combined durations as required. This information can be used for more nuanced interrogation of other datasets to obtain representative values based on the spatial precision of the estimate.

### Latent estimates

Estimates of latent locations can be obtained, representing the intermediate positions between those explicitly measured. These represent each period between Argos locations or times between each twilight for archival tags: in general they represent periods between those of (primary) data collection relevant to location estimation. Latent estimates may also be summarized as a mean and CI, and used to provide estimates of the full path between individual time steps. The density of intermediate locations provides a model of the possible range of the track, similar in intention to the spatial smoothing mechanisms employed in other studies.

While direct estimates are constrained by likely movement regimes as well as the available data, the latent estimates represent the residual possible movement in-between.

Unlike some studies using techniques that require subsequent clipping [14], [25], time spent estimates can be made without spurious presence on land or other out-of-bounds areas. Also, there is a more realistic probability transition from land to marine areas even for complexly shaped coastlines.

The use of latent estimates utilization distributions is better than either cell gridding or kernel density as there is no dependence on the choice of grain size or kernel. The final step to quantize values into a density grid can be done directly from the posterior, without intermediate processing.

### Combinations

The structure of our estimates enables us to combine estimates from different animals for spatial measures of resource usage. This may be done for arbitrary time periods and groups of individuals. Also raw coordinates may be projected for summaries based on an appropriate coordinate system for particular groups or areas of interest.

### Updating the models

Time spent maps and track summaries (mean and CI values) were generated by summarizing the posterior for each example. The intermediate locations represent the ‘full path’ and hence are appropriate for time spent maps and similar spatial summaries. The direct locations are estimates for each time step from the raw location data - individual twilights for the archival tag, Argos times for the satellite tag. Interrogating individual x or z estimates provides feedback on the performance of the model run that may be used to identify problems or areas that require improvement. An example of this feedback was discussed with Figure 4 where we see how the movement model requires an improved implementation for the satellite tag. This is one of the most powerful aspects of our approach, more important than the results presented here as it provides a foundation from which remaining problems with location estimates may be identified and related to deficiencies in source data, model specification or model assumptions.

Other studies have successfully applied Bayesian methods to tracking problems with similar success [11], [51], but applied only to pre-derived location estimates, and it is not clear how archival tag data could be incorporated in such an approach. The quantities of data involved and the non-linear complexity of the models involved are difficult to implement with more efficient statistical sampling regimes such as Gibb's sampling. Our approach enables the use of the raw archival tag data and incorporation of independent environmental databases. High quality location methods such as satellite tracking can also benefit from our approach. For example: similar to the satellite example presented here, [52] also report dealing with large numbers of Argos locations that were clearly deficient as they place marine animals on the land. Our approach allows the systematic use of the appropriate coastline to data account for this inconsistency.

The advantages of our approach are relevant to all users of tracking data including tag manufacturers, ecological researchers and environmental decision makers. The key benefits are:

1. A convenient mechanism for separating large complex problems into manageable components, enabling the use of all available information sources.
2. Obviously incorrect locations are avoided, and when data are absent or of poor quality the estimates will have a lower precision.
3. Estimates are continuous in the posterior and may be summarized as required, rather than being discretized or otherwise simplified.

While we have illustrated our approach using seals, these techniques clearly have broader implications for the tracking of other species and other tagging methods. This approach to location estimation better enables multi-species ecosystems comparisons irrespective of the methods used to collect data. A particularly important area of application is in fishery studies, which have large quantities of archival tag data e.g. [53] and [12], or satellite data e.g. [25]–[27]. The improvement of location estimation will enable further research aimed at relating fisheries management to that of other marine species and processes.

While our approach can provide location estimates with confidence intervals based on the data model, there remains the need for independent validation of the techniques with known locations. The assessment of accuracy of these techniques is crucial to their use, and opportunities exist with double-tagging experiments, recapture studies and experimental validation.

The relationship between tag-measured temperatures in near-surface waters and remotely sensed surface temperature remains largely unexplored in animal tracking studies [54]. This is due to the discrepancy between traditional physical oceanographic interests and those of biological studies. Access to hierarchical datasets of SST [18], models of surface and at-depth water temperature and sources of higher quality local environmental data will improve the contributions from this auxiliary information. A more detailed approach would match auxiliary data values in a probabilistic sense similar to methods employed by [12], enabling the application of distributions to account for error in all measurements.

The use of depth and temperature at depth also remains a largely unexplored aspect, no further work has been published since [4] and [41]. The utility of this data source obviously depends on the environment visited and the animal's diving behavior, but also highlights the breadth of opportunities that are available for various species.

Many of our implementation decisions have been deliberately based on simplistic, first-pass practicalities in order to demonstrate the generality of our approach to a wide range of problems. The application of MCMC demands careful diagnosis of model convergence [55] and we have omitted this important but onerous aspect from the present work in order to focus on the primary goal of integrating all the available data. While our movement model is flexible it does not account for movement regimes that are auto-correlated or seasonal. Auto-correlation of speed is recognized as an important aspect of modelling movement, also missing from our initial implementation. For example, in both examples we have assumed that the successive 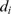 are independent. However, we can model serial correlation in the track by choosing the joint distribution of distances so that successive 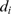 are correlated. The impact of a variety of correlation models could be explored [11], [56].

In this study we applied a single scheme to the derivation of location estimates from two very different tracking datasets. Each dataset was composed of separate sources of information integrated using our four-part approach. This was used to derive location estimates from raw archival tag data, as well as from pre-derived location estimates from a satellite service. In each case, where limitations from a particular source could have produced problematic estimates, this was augmented by the strengths of others.

This method is clearly practically applicable to the real-world problem of analyzing behavior from many large archival tag datasets employed by marine animal studies, and is appropriate for the tracking data from many species. It is also useful for applying behavioral constraints to the latent aspects of nearly error-free location estimation such as GPS.

## Supporting Information

Figure S1 Argos full path estimates with raw location track. Animation of full path estimates constructed from the posterior for *z*. The sequence consists of a rolling 2 day window for every 10 hour interval of the tagging period. The matching sequence of original raw Argos locations is overlaid as a line.
(0.47 MB GIF)
