# Supplementary figures and images for: Bayesian Estimation of Animal Movement from Archival and Satellite Tags
Source: PLoS One. 2009 Oct 13;4(10):e7324. doi: 10.1371/journal.pone.0007324 (PMC2758548; doi:10.1371/journal.pone.0007324)

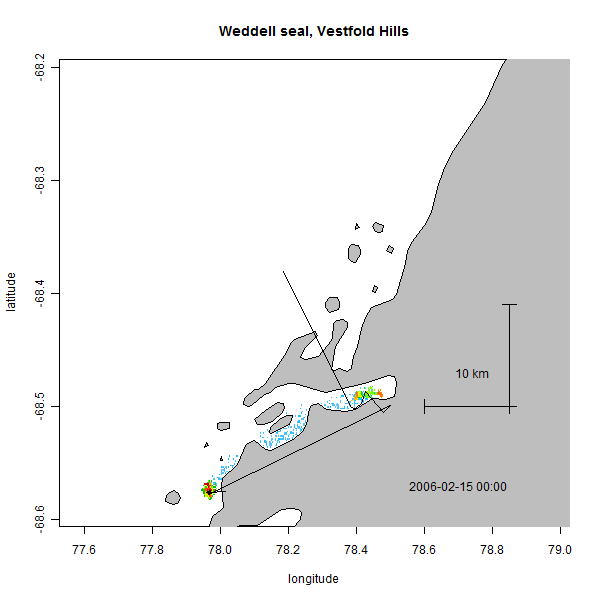

Supplement: Figure S1 — Argos full path estimates with raw location track. Animation of full path estimates constructed from the posterior for z. The sequence consists of a rolling 2 day window for every 10 hour interval of the tagging period. The matching sequence of original raw Argos locations is overlaid as a line. (0.47 MB GIF) [file pone.0007324.s001.gif]
